# A content analysis of depression-related discourses on *Sina Weibo*: attribution, efficacy, and information sources

**DOI:** 10.1186/s12889-018-5701-5

**Published:** 2018-06-20

**Authors:** Jiabao Pan, Bingjie Liu, Gary L. Kreps

**Affiliations:** 1 The School of Culture and Media, Central University of Finance and Economics, No. 39 Xueyuan South Road, Haidian District, Beijing, 100081 China; 2 Donald P. Bellisario College of Communications, The Pennsylvania State University, 115 Carnegie, University Park, PA 16801 USA; 3 Department of Communication, George Mason University, Robinson Hall A339, MS 3D6, Fairfax, VA 22030-4444 USA

**Keywords:** Depression, Attribution, Efficacy, User-generated content

## Abstract

**Background:**

Depression is a mood disorder that may lead to severe outcomes including mental breakdown, self-injury, and suicide. Potential causes of depression include genetic, sociocultural, and individual-level factors. However, public understandings of depression guided by a complex interplay of media and other societal discourses might not be congruent with the scientific knowledge. Misunderstandings of depression can lead to under-treatment and stigmatization of depression. Against this backdrop, this study aims to achieve a holistic understanding of the patterns and dynamics in discourses about depression from various information sources in China by looking at related posts on social media.

**Method:**

A content analysis was conducted with 902 posts about depression randomly selected within a three-year period (2014 to 2016) on the mainstream social media platform in China, *Sina Weibo*. Posts were analyzed with a focus on attributions of and solutions to depression, attitudes towards depression, and efficacy indicated by the posts across various information sources.

**Results:**

Results suggested that depression was most often attributed to individual-level factors. Across all the sources, individual-level attributions were often adopted by state-owned media whereas health and academic experts and organizations most often mentioned biological causes of depression. Citizen journalists and unofficial social groups tended to make societal-level attributions. Overall, traditional media posts suggested the lowest efficacy in coping with depression and the most severe negative outcomes as compared with other sources.

**Conclusions:**

The dominance of individual-level attributions and solutions regarding depression on Chinese social media on one hand manifests the public’s limited understanding of depression and on the other hand, may further constrain adoption of scientific explanations about depression and exacerbate stigmatization towards depressed individuals. Mass media’s posts centered on description of severe outcomes of depression without suggestions of solutions’ effectiveness, which may induce more anxiety among depressed individuals. Campaigns promoting comprehensive understandings about depression and popular works translating scientific findings on depression to the public are called for.

## Background

Depression is a common yet severe mood disorder with holistic impact on individuals including their mood, perceptions, cognitions, and daily activities such as sleeping, food intake, working, etc. [1, 2], affecting more than 300 million people worldwide [3]. In China, more than 54 million people (4.2% of the population) were suffering from depression by 2015 [4]. Despite the increasing prevalence of depression in China, compared to other countries, the rate of diagnosed depression is relatively low [5, 6], suggesting a collective denial and under treatment of depression as a disease [6].

Although development of depression is a complex process with biological, psychological and social factors involved [7], public’s understanding about it is often simplistic. In a survey conducted with citizens of three major cities in China, most respondents thought symptoms of depression could be relieved by themselves without professional help and considered “talking to families and friends” and “resting more” as sufficient solutions [8]. The lack of scientific understanding of depression contributes to stereotyping and stigmatization towards depressed individuals. In an online survey, nearly half of the Chinese participants described depressed individuals with terms like “pessimistic suicide” or “with rabid, aggressive and weird character” [9]. Stigma brings shame and guilt to both the depressed individuals and their families, further discouraging them to reach out for professional help [10, 11].

During recent years, social media are playing an increasingly important role in disseminating knowledge about health issues and shaping public attitudes with user-generated content (UGC) [12]. Compared with mass media, research suggests that discussions on mental health issues on social media are more objective and more educational [13], which warrants a closer examination of social media content about mental health [14]. However, to our knowledge, investigations of depression-related discourse on Chinese social media are still rare [15–17]. Against this backdrop, the current study aims to study how depression is represented on *Sina Weibo*, the most popular social media platform and venue for civic engagement and activism in China today [18], with a focus on users’ understandings of depression in terms of its causes, solutions, and their general attitudes to depressed individuals.

### Attributions, solution, and efficacy

#### Social stigma and attribution

A major obstacle for depressed individuals when reaching out for help is the stigma associated with depression. Research has found that depressed individuals are often associated with negative labels such as “weak mind,” “drama,” and “psycho” in China [19]. An analysis of 15,879 depression-related posts on *Sina Weibo* found that 6.09% of the posts indicated stigma [17].

Research suggests stigma and blame on individuals depend on what factors the outcome is attributed to [20]. Attribution is a process of causal analysis of behaviors and events. Different typologies of attribution exist such as internal-external [21], controllable-uncontrollable, stable-temporary, global-specific attribution [22], etc. which are applicable to different research contexts.

When it comes to stigma, the controllable-uncontrollable typology is the most relevant [20]. Research suggests that the public is more tolerant with diseases caused by uncontrollable factors than those caused by factors that can be controlled by individuals such as sexually transmitted diseases or tobacco use related diseases [23]. Unfortunately, mental diseases, as compared with physical diseases, are more subject to attribution to controllable factors, and as a result, people with mental diseases are often stigmatized [23]. As Weiner suggested, if one’s depression is believed to be caused by his/her own doings instead of environmental factors, people tend to hold negative attitudes towards the depressed [24]. Even portraying mental illness as a physical malfunction of the brain can reduce the blame placed on patients because it is considered as out of individual’s control [25].

Wang and Liu analyzed depression-related posts generated by top 10 mainstream media organizations and public opinion leaders and their followers’ responses to them on *Sina Weibo*. By putting attribution into four categories, namely biological/genetic (i.e., family genes), environmental (i.e., accidents, work issues, and changes in the life cycle), parental (i.e., parental mistakes or abuse) and personal (i.e., character flaws), they found that when these influential users claim relatively uncontrollable factors as causes of depression, such as biological/genetic and environmental factors, their followers’ comments contained less stigmatization [15, 16]. Therefore, to understand the root of stigmatization towards depression, it is important to examine how controllable are the factors that depression is attributed to by depressed individuals.

### Solution oriented attribution

Depression is not only a negative private experience but also a public health issue calling for a solution. Besides the controllable-uncontrollable dimension, whether it is attributed to individual-level or institutional factors is more relevant in terms of determining whose responsibility it is to solve the problem, which has important implications on health policies [26, 27], practices, and public opinions [28]. Attribution to biological and medical factors suggests changes to make in the scientific and medicine domain. If it is the depressed individuals who are primarily responsible for their suffering, arguably, they should take actions such as self-help. Factors related to parental and other close relationships suggests changes made by individuals who care about depressed people. If development of depression is attributable to societal factors, then it implies that it is the public sectors’ responsibility to solve the problem, for instance, by making new policies or improving medical care systems [29]. Therefore, when looking at attribution of depression in social media discourse, it is necessary to generate a finer taxonomy of agency locus that is beyond the controllable-uncontrollable dichotomy.

### Efficacy

Although attribution to uncontrollable and external factors alleviates blame on and the responsibility of depressed individuals, it suggests little can be done by depressed individuals [15, 30]. Fortunately, many social media users actively share information and their first-hand experiences regarding causes, symptoms and diagnosis, prevention and treatment of diseases [31]. A recent analysis of 2000 depression-related tweets on Twitter found supportive information prevails such as encouraging words, information about depression prevention and how to help depressed individuals [14], which might enhance individuals’ efficacy and confidence in dealing with depression [32].

As suggested by the Extended Parallel Process Model [33], enhancing the perception of response efficacy, i.e., effectiveness of a recommended strategy, empowers individuals and better scaffolds them in coping with diseases. Highly effective method is expected to bring depressed individuals with more hope and confidence. Research found that advice is mostly likely to be taken when its usefulness is explicitly addressed [32]. Therefore, UGC on depression will be more supportive if it suggests positive outcomes of using certain strategies.

The severity of disease outcomes also has implications on one’s efficacy. Highlighting severe outcomes of a disease, i.e., fear appeal, is often employed as a strategy to increase individuals’ awareness of risks [33–35]. Content analysis of tweets with the hashtag #MyDepressionLooksLike on Twitter reveals themes of negative outcomes of depression such as dysfunctional thoughts, lifestyle challenges, social struggles, apathy and sadness, and about 5% of the content is about self-harm and suicide [36]. Another analysis of depression-related content on *Pinterest* found 10% mentioning suicide and 7% mentioning self-injury, with coping strategies rarely shared [37]. However, mentioning severe outcomes of depression such as suicide or serious injuries without suggesting solutions will not motivate individuals to take any actions but only induce more anxiety among them [33].

All these potential influences of UGC about depression warrant closer investigation of communication about depression flowing on social media. However, little is known about the situation of Chinese social media. Although Wang and Liu [15, 16] set a good start, they only focused on content generated by public opinion leaders and mass media users, but left out original contents generated by other sources, such as ordinary individual users, the content from whom directly reveals the public’s understanding of depression, and other agencies with more direct influence on and relevance to public policy making such as governments. In addition, different information sources hold different positions on the political economy spectrum, and arguably, may express different interpretations of and attitudes to depression, and also have different impact on public opinions and policy making [15, 38]. To gain a more comprehensive understanding about depression-related discourses on Chinese social media, the current study takes all the individual and intuitional users in to account, with the focus on attribution, solution and efficacy related to depression as revealed in their posts. We proposed the following research questions:

RQ1. What are the a) attribution frames and b) solution frames about depression, and c) how is the efficacy in dealing with depression suggested in depression-related discourse on *Sina Weibo*?

RQ2. What is the relationship between information sources and a) the attribution frames and b) solution frames about depression, and c) the efficacy in dealing with depression suggested in depression-related discourse on *Sina Weibo*?

## Method

### Sampling

*Sina Weibo* allows researchers to retrieve messages posted during a specified period through keyword searching. With “depression” as the keyword, we retrieved posts for 3 years from January 1st, 2014 to December 31st, 2016. We followed the procedure of systematic random sampling by selecting the first post displayed on every two pages (20 posts or less per page). If the first post on a page was not about human depression, we moved to the second post, until reaching a qualified message. In total, we got a sample of 902 posts about depression.

### Coding scheme

With a focus on the attribution of and solution to depression, and efficacy in coping with depression, we developed a coding scheme with the following variables. To be noted, one post might imply multiple types of attribution and solution, therefore, more than one values of each variable might be ascribed to a post.

#### Information source

1. Ordinary *Weibo* users; 2. Health experts (including experts in psychology and health) and health organizations (including hospital, counselling office, etc.); 3. Elites in other domains (individual accounts with *Sina Weibo*’s verification, like Twitter’s verified account); 4. State-owned media organizations; 5. Market-orientated media organizations; 6. We-media [39] or citizen journalists; 7. Corporations; 8. Government and government controlled organizations; 9. Academic organizations; 10. Social organizations or groups, including non-governmental organization (NGO), non-profit organization (NPO) and unofficial social groups.

We adopted the coding scheme for attribution and solution frame from Wang and Liu [15, 16].

#### Attribution frame and solution frame

1. Biological and medical factors/solutions; 2. Personal factors/solutions, e.g., self-regulation and adjustment; 3. Factors/solutions related to parental and other close relationships; 4. Structural-level factors/solutions, e.g., social structure, community and public policy; 5. Not mentioned.

#### Attitude towards depressed individuals

Valence of the sentiment in each post directly ties to stigmatization towards depressed individuals. We coded attitudes towards depressed individuals as either 1. Positive; 2. Neutral; or 3. Negative.

#### Coping strategies

Categories include 1. Medical care; 2. Social support; 3. Self-regulation and adjustment and 4. Not mentioned.

#### Response efficacy

Posts were categorized into one of the following categories according to the outcome of using certain coping strategies, 1. Low response efficacy (i.e., suggesting depression can hardly be cured, or depressed individuals still commit suicide after getting treatment); 2. High response efficacy (i.e., suggesting depression is curable, or states of depressed individuals improve after adopting certain strategies); and 3. Not mentioned.

#### Severity

Severity of depression outcomes was categorized as 1. High severity (i.e., with death or serious physical hurt as outcomes); 2. Moderate severity (other outcomes that are less severe than death or physical injury), and 3. Not mentioned.

#### Hyperlink

Considering the 140-character limit for each post on *Sina Weibo*, richer information is typically delivered with hyperlinks embedded in the posts. Therefore, we consider the presence of hyperlinks and the content they direct to as important indicators for information sharing. Specifically, posts were coded into the three following categories, 1. None; 2. News reports; 3. Health-related information (including one’s own storytelling, coping strategies, diagnosis, etc.).

### Intercoder reliability

Two authors of this study first independently coded about 11% (100 posts) randomly selected from the sample. Intercoder reliability was calculated using Holsti’s method, ranging from .80 to 1.00 on all coding categories, indicating acceptable levels of agreement between coders [40]. After resolving disagreements through discussion, the rest of the posts were divided between the two authors and coded independently.

## Results

### Overall findings

As shown in Table 1, among the 902 posts in our sample, 58.31% were from ordinary *Weibo* users, followed by elites from domains other than health (9.87%) and health experts and organizations (7.10%).

**Table 1:** Source, attribution frame, efficacy and severity in depression posts

	Coding Categories	# of posts	Percentage
*Sources*			
	Ordinary users	526	58.31%
	Opinion leaders in other domains	89	9.87%
	Health expert and health organizations	64	7.10%
	Market orientated media	63	6.98%
	State-owned media	51	5.65%
	We media	32	3.55%
	Government	26	2.88%
	Corporations	25	2.77%
	Academic organizations	14	1.55%
	Social groups	12	1.33%
*Attribution Frame*			
	None	520	57.65%
	Personal	246	27.27%
	Biological and medical factors	97	10.75%
	Social structure, community and public policy	56	6.21%
	Parental and close others	54	5.99%
*Solution Frame*			
	None	452	50.11%
	Self-adjustment	222	24.61%
	Biological and medical factors	177	19.62%
	Parental and close others	88	9.76%
	Social structure, community and public policy	26	2.88%
*Attitude towards people with depression*			
	Positive	109	12.08%
	Neutral	695	77.05%
	Negative	98	10.86%
*Coping Strategies*			
	None	475	52.66%
	Self-adjustment	219	24.28%
	Medical care	171	18.96%
	Social support	86	9.53%
*Response Efficacy*			
	Not mentioned	622	68.96%
	High	221	24.50%
	Low	59	6.54%
*Severity*			
	Not mentioned	475	52.66%
	Moderate level	219	24.28%
	High level	208	23.06%
*Information Sharing*			
	None	727	80.60%
	Health-related information	107	11.86%
	News reports	68	7.54%

As for the patterns of attribution, about half of the posts (57.65%) did not make attributions about depression. Among those posts with attributions, most of them (64.40%, i.e., 27.27% of all the posts) attributed depression to individual-level factors such as personality and life styles. For example,

*“Seeing difficulties with pessimism, feeling helpless and desperate, is the fundamental cause of depression.”*

*“Thinking of one of my friends…She was quite impressive, she published her work and got paid when she was in the middle school. But probably because of having read too much, she got a bit odd, and because she was very pretty and sort of aloof, she didn’t get along with other girls in our class, so she developed depression gradually.”*

The second most frequently made attribution was biological and medical attribution (10.75% of all the posts). For example,

*“#bright depression# it is genetic. It is important for you to change. Actually, you are already determined.”*

*“…If the endocrine process also goes irregular plus fatigue, it is very likely to develop depression.”*

Only 6.21% of all the posts attributed depression to social-level factors such as the demographic categories one belongs to as causes for depression. For example,

*“Chinese American has the highest rate of depression among all the Asian Americans. Female Chinese American aged 15-24 has the highest rate of suicided among all the Americans.”*

*“According Guangzhou Daily, there is a huge gap between the have and the have-not. As research shows, the second generation of the rich is even more subject to depression and anxiety than the second generation of the poor.”*

Attribution to parental and close others was found least frequently (5.99%) among all posts. For example,

*“Every time I go home, my depression gets worse.”*

*“I was shocked when I knew one of my students got depression, and worried at the same time. She used to be a cute gentle girl, but now got depression because of her family.”*

Accordingly, the pattern in the solution frame is largely consistent with that of attribution. Compared with attribution, users were more likely to mention solutions to depression on *Weibo* (49.89% mentioned solution among all the posts). Self-adjustment and medical help were the first and second frequently mentioned solution. An example of self-adjustment was.

*“I won’t take any antidepressants because its side effects will make me suffer more. I will fight this devil with my strong will!”*

*“There is nobody and no cure to help you. The only way is self-adjustment.”*

Among all the posts, 9.76% of suggested the importance of support from families and friends as solutions to depression. For example,

*“Please take care of depressed individuals around you. Call 6666.”*

*“Please care more about people with depression around you. Call them more often, bring them to parties, take them to the nature, send them plants and flowers.”*

Users mentioned making changes in society or public policy as solutions the least. For example,

*“More than half of depressed individuals feel they cannot pay full attention and have low working efficiency, and therefore, have to take sickness leave. 53% of the managers hope the government can make better policies to protect people with depression.”*

*“The girl who lacks correct self-knowledge just left like this. It is the responsibility of the entire society! Mental health education is more than writing microblogs. We need the entire society and all platforms to rectify the values of the young!”*

The most often mentioned coping strategies were individual adjustment, without turning to external help, such as changing daily routines and ways of thinking (24.28% of all the posts). For example, advice such as “taking more rest/exercise,” “eating more fish/banana/apple” fell in this category. Following that 18.96% suggested seeking professional help, including “psychological counseling” “medication” or general saying like “going to hospital.” Only 9.53% highlighted the importance of social support in dealing with depression. For example,

*“I was depressed once. The persistent caring from teachers and friends made me learn how to accept others and smile, rather than trap myself in a dead end.”*

Among all the posts in the sample, 31.04% indicated the response efficacy of certain coping strategies. High-efficacy posts, for example, were like “though spring is marked by high rate of developing depression, people can still cope with that with slight adjustment” or emphasized depression as preventable and curable. Low-efficacy posts were like “depression is a serious mental problem that technology and medicine can’t solve it completely.” Overall, high-efficacy posts (24.50% of all the posts) were more common than low efficacy (6.54% of all the posts).

Nevertheless, among all the posts, 47.34% mentioned the outcomes of depression, half of which suggested that depression might lead to serious individual and social outcomes, such as suicide and murder. A total of 622 out of the 902 posts (68.96%) indicated severity of depression, including bad mood in daily life, self-harm, suicide and murder, only 221 (24.50%) included information suggesting high effectiveness of certain solutions.

Sharing information with a link or a video was not common on *Weibo*. Among the 902 posts analyzed, 727 (80.60%) of them did not include hyperlinks to additional information. About 11.86% of all the posts contained hyperlinks to information about personal experience with depression, depression diagnosis and strategies to alleviate symptoms, knowledge about depression, and common misunderstandings about depression and depressed individuals, which typically took the form of a personal blog or a documentary about depression. In addition, 7.54% of the posts shared links to news articles or depression-related news programs.

[Table 1. Source, attribution frame, efficacy and severity in depression posts].

### Source and attribution

Ordinary users tended not to talk about how people developed depression (only 37.07% of all the posts by ordinary users mentioned attribution). Instead, the majority of their posts centered on describing their current conditions and help seeking. State-owned media and academic organizations mentioned causes the most.

Almost all the source categories, including ordinary users, opinion leaders in other domains, media, government and corporations, attributed depression most to individual-level factors. Compared with commercial media (23.81%), state-owned media organizations were more inclined to make attributions to individual-level factors (43.14%) and are the most likely to make individual-level attributions among all the source categories. Compared with other attribution frames, academic organizations, health experts and organizations were more inclined to make attributions on the biological and medical level (academic organizations: 50.00%; health experts and organizations: 28.13%). Among all the attribution frames, societal-level attribution was the most often used frame by NGO and NPO, we media and citizen journalists (NGO and NPO: 25.00%, while 50% didn’t mention any attribution; we media and citizen journalists: 15.63%, while 62.50% didn’t mention any attribution).

### Source and solution

The pattern we found for solutions to depression across sources and solutions is not quite consistent with that for attribution. Overall, government and health experts and organizations tended to mention more about solutions to depression. While media (including commercial media, state-owned media, and we-media) attributed depression more to individual-level factors than biological and medical factors, the most mentioned solutions by them were medical treatment or psychotherapy. Consistent with their attribution pattern, health experts and organizations were more likely to suggest solutions on biological and medical levels among all the sources categories, followed by academic organizations (health experts and organizations: 48.44%; academic organizations: 35.71%).

Governments and commercial corporations were more inclined to suggest self-adjustment as the solution (governments: 46.15%; commercial corporations: 44.00%). Although all sources relied on societal-level solutions the least, government and unofficial social groups tended to mentioned more about social or common efforts, as compared with other sources.

### Source and efficacy

In terms of the efficacy of coping with depression, media (including commercial media, state-owned media), we-media and ordinary *Weibo* users were less likely to mention efficacy information. About 70% of the posts by them did not include any information suggesting the effectiveness of any solutions. Although high efficacy was much more common than low efficacy among posts from all sources, posts by media and ordinary users were more likely to reveal a low efficacy compared with other sources. For example, media told news stories such as depressed individuals committed suicide while receiving medical treatment, and ordinary people expressed very limited confidence in controlling their own depression. Among all the posts by governments, corporations, academic organizations and social groups, none of them indicated low efficacy.

In terms of severity of depression outcomes, about a half of the posts talked about the outcomes of depression. Government and media (including both the commercial media and state-owned media) were the sources mentioning suicide and murders as the outcomes of depression the most. State-owned media were especially open to mention the outcomes of depression (58.82%) as compared with other sources. Corporations, ordinary Weibo users, we media and citizen journalists were the top three sources indicating low-level severity, such as bad mood, moderate negative impact on work and daily life, etc.

Among the 107 posts sharing depression-related information, ordinary users (*n* = 51, 47.66%) and psychology and health experts and health organizations (*n* = 22, 20.56%) made the major sources.

### Source and attitudes towards depressed individuals

In terms of the attitudes expressed towards depressed individuals, the majority of the posts (77.05%) held a neutral attitude, 12.08% held a positive attitude, and 10.86% held a negative attitude. Compared with other sources, ordinary users were more likely to reveal their attitudinal positions explicitly (either positive or negative). Government (15.38% negative, no positive) and media (14.04% negative, while 10.53% is positive) were the most inclined to express a negative attitude towards depression and depressed individuals. In contrast, we media and citizen journalists and unofficial social organizations were most likely to express a positive attitude. Among the posts by we-media and citizen journalists, 21.88% revealed a positive attitude and 6.25% showed a negative attitude. Among the posts by unofficial social organizations and groups, 16.67% held a positive attitude and 8.33% expressed a negative attitude.

## Discussion

The current study aims to investigate depression-related discourses in China formed on a popular social media platform, *Sina Weibo*, with its focus on examining attribution of depression, solution to depression, and efficacy as suggested in the messages, and how their patterns vary across different sources. Attribution of depression is important in that it suggests the locus of the agency and responsibility, which has implications on understanding depression, attitudes to depressed individuals, and intervention. The development of depression is attributable to multiple factors that might or might not be subject to individuals’ control. However, as the results revealed, individual-level attributions were the most often applied frame in general. Such an attribution implies that the crux of depression is located within individuals themselves and individuals should be responsible for their suffering. Following this assumption, depressed individuals are those who “make” themselves depressed, whereas others are those who succeed in avoiding depression. Such dichotomization suggesting depressed individuals are “soft minded” and “deserve the suffering” might contribute to stigmatization and discrimination towards them.

Taking information sources into account, the individual-level attribution frame was the most often used frame for ordinary users, elites from other domains, market-oriented media, governments, corporations, and especially state-owned media, suggesting that the general public and organizations without health expertise are more likely to ignore the social and biomedical factors related to depression. In contrast, health experts and organizations, and academic organizations more often attributed depression to biological and medical factors. Whereas citizen journalists and unofficial social groups tended to appeal to societal-level factors as explanations.

In the current study, attributions made by each type of sources were largely consistent with their political standings. Grassroots individuals and social groups aim for progress on the societal level while health and academic institutions aim to facilitate scientific progress. Attributions made by established institutions were also consistent with their standings that the social structure functions well in large and individuals can adjust themselves to fit in.

Severity of depression’s outcome, if depicted as too serious with limited efficacy suggested in the message, may result in more anxiety among depressed individuals but not motivate them to take actions [33]. A total of 69% posts (622 out of 902) mentioned high or moderate level of severity of depression’s outcome, but only 221 posts contained information suggesting high response efficacy.

Among all the sources, governments and mass media were the most likely to mention suicide and murders as the outcomes of depression. While governments also highlighted that depression is preventable and curable, posts by mass media most often suggested limited effectiveness of solutions, followed by ordinary users.

As found in our study, 77.05% of the posts held a neutral attitude towards depressed individuals. Among the posts explicitly expressing clean-cut attitudes, the percentage of posts with positive and that with negative attitudes were roughly equal. It seems that health experts and organizations, unofficial social groups and organizations, and we media/citizen journalists were more likely to hold a positive attitude towards depressed individuals, while market-oriented media, academic organizations, and governments tended to express negative attitudes. In other words, people with expertise in depression and the grassroots organizations treat depressed individuals more positively than the governments and market-oriented media. Depending on which source people rely on more in getting depression-related information, their attitudes to depression might shift accordingly.

### Media organizations on social media

Mass media organizations are often deemed of high credibility especially in covering public issues [41] and have broader reach to the audience as compared with other sources. As found in our study, suicide, serious injuries, and murders, were the most frequently reported outcomes in posts by mass media, consistent with previous findings in other countries about mass media’s representations of mental disorders [42–45], which may contribute to stereotyping depressed individuals as dangerous “the insane.” In addition, compared to other sources, posts by mass media indicated lower response efficacy. This pattern is also consistent with previous findings with content analysis of news reports on other health-related issues on Chinese media, such as lung cancer and breast cancer [46, 47]. Among all the posts suggesting low efficacy by mass media, the majority (53.33%) were about medical treatment. In other words, professional help was most associated with low effectiveness in mass media’s posts, which may discourage people in need from seeking professional help. By reporting many serious outcomes of depression but not scaffolding with efficacy, Chinese media organizations seem to be playing a depressing role on social media.

In the current study, we found a discrepancy between the attribution frame and solution frame in posts by mass media. Although mass media accounts highlighted individual factors causing depression such as their personalities and life-style rather than biomedical ones, when it comes to solutions, they talked more about medication and psychotherapy than self-adjustment across all three years. With a closer look at the data, we found that the inconsistency was related to the suicide of *Renliang Qiao* in September 2016, a famous Chinese actor and singer who had been suffering from depression. After his death, posts by mass media contained more information about coping strategies, especially seeking professional help. However, in 2014 and 2015, mass media mentioned individual-level solutions such as self-adjustment more often than biomedical ones, consistent with their pattern of attribution. What we found here also corroborates a finding from a survey that Chinese journalists had very limited knowledge about mental health such that only 10% of the journalists thought it was necessary to seek medical treatment for those with serious mental illness [48].

Findings in this study also suggest the esteem threat on depressed individuals. As found in past studies [15, 16], given the negative outcomes of depression and the long existing stigma associated with mental illness in China, depressed individuals are often stigmatized and otherized in public discourses. Some have already internalized such stigma, hold negative self-evaluations, and feel ashamed of themselves as being “weak-minded,” as suggested by some posts by depressed individuals in our sample.

Fortunately, among the 902 posts, we found 107 described one’s subjective experience with depression or other’s life stories related to depression, in the form of hyperlinks embedded in the posts. By sharing one’s own or others’ experience, depression is legitimized as a common experience, which is expected to protect depression patients’ self-esteem [49] and provide depressed individuals a sense of belonging and a sense of community. Those with limited understanding about depression may also learn about the patients’ perspectives so that they can provide social support of better quality.

### Limitations

The present study is not exempt from limitations. First, we only analyzed posts during three years, from 2014 and 2016. Within such a limited time scheme, we cannot conclude about the pattern shift in depression-related discourses over time. In the future, we would like to expand the population studied to posts from the year of 2009, when *Sina Weibo* was first launched. In addition, a parallel analysis on mass media’s portrayal of depression would also help better understand the interaction between traditional media and social media in shaping the public discourse about depression.

Second, results reported here are mainly descriptive. It is also important to know the direction of social influence within a social network. Social media have equalized the power distribution in terms of communication to some extent [50]. The direction of social influence might not be only from traditionally defined “opinion leaders” to “followers.” Stories of ordinary users, especially those with depression and those with first-hand experience with depression, may have impact on opinion leaders, organizations, and mass media. A study of that kind might provide suggestions on social media designs to augment impact of grassroots and empower depressed individuals, reducing the otherness sustained by existing discourse and creating a more tolerant and understanding cyber social environment.

Third, since we only have access to public posts on *Weibo*, our analyses did not encompass content generated in private. Therefore, we are unable to speculate the patterns and dynamics of the private discourse about depression. Future research might further explore on this topic by investigating interpersonal communication related to depression.

## Conclusions

In conclusion, with the content analysis of depression-related discourses generated by social media users, we found depression still most often attributed to factors controllable by the depressed individuals. Mass media are especially prone to use such an attribution frame in their posts on social media. Moreover, discourses generated by mass media imply low efficacy in coping with the severe outcomes of depression. Although grassroots users and organizations are coming up with alternative attributional and solution frames, considering the authority status of mainstream mass media in shaping public opinions and its greater reach on social media platforms, the current depression-related discourses formed on Chinese social media are concerning. Campaigns promoting alternative and more sophisticated understandings about depression are needed and media institutions should aim to better translate scientific findings on depression to the public.
